# Description of the karyotypes of Stejneger's beaked whale (*Mesoplodon stejnegeri*) and Hubbs’ beaked whale (*M. carlhubbsi*)

**DOI:** 10.1590/1678-4685-GMB-2016-0284

**Published:** 2017-10-02

**Authors:** Nozomi Kurihara, Yuko Tajima, Tadasu K. Yamada, Ayaka Matsuda, Takashi Matsuishi

**Affiliations:** 1 Faculty of Agriculture, Utsunomiya University, Utsunomiya-shi, Tochigi prefecture, Japan; 2 Department of Zoology, National Museum of Nature and Science, Tsukuba-shi, Ibaraki prefecture, Japan; 3 Graduate School of Fisheries Sciences, Hokkaido University, Hakodate-shi, Hokkaido, Japan; 4 Faculty of Fisheries Science, Hokkaido University, Hakodate-shi, Hokkaido, Japan

**Keywords:** karyotype, chromosome, Mesoplodon stejnegeri, Mesoplodon carlhubbsi

## Abstract

The genus *Mesoplodon* (Cetacea: Odontoceti: Ziphiidae) is one of the few cetacean genera with the karyotype 2n = 42. The 2n = 42 karyotype of *M. europaeus* and *M. carlhubbsi* is largely consistent with the general cetacean karyotype 2n = 44, although other 2n = 42 karyotypes do not exhibit clear homologies with the general cetacean karyotype. Therefore, the chromosomes of *Mesoplodon* species may be the key to understanding cetacean karyological evolution. In the present study, the male karyotypes of *M. stejnegeri* and *M. carlhubbsi* were examined. In both species, the diploid number of the male karyotype was 42. Both species had the following characteristics: 1) a huge subtelocentric X chromosome with a large C-block; 2) a small metacentric Y chromosome; 3) nucleolus organizer regions (NORs) in the terminal regions of a large autosome and one or two small metacentric autosomes; 4) small metacentric autosomes; 5) large submetacentric and subtelocentric autosomes; 6) less accumulated C-heterochromatin in the centromeric region; and 7) heteromorphism in C-heterochromatin accumulation between homologues. Characteristics 1 and 3 are peculiar to only the karyotypes of *Mesoplodon* species, whereas characteristics 4, 5, 6, and 7 are also found in the species with the general cetacean karyotype 2n = 44.

Two diploid chromosome numbers are known in the order Cetacea: 2n = 44 and 2n = 42 (Árnason, 1974). Most cetaceans have the karyotype 2n = 44, and many authors have pointed out the uniformity in chromosome morphology and banding pattern among cetaceans with this karyotype (*e.g.*, Árnason, 1974, 1980; Duffield *et al.*, 1991). The karyotype 2n = 42 has been described in only seven species: *Eubalaena glacialis* (Pause *et al.*, 2006), *Balaena mysticetus* (Jarrell, 1979), *Physeter macrocephalus* (Árnason and Benirschke, 1973), *Kogia breviceps* (Árnason and Benirschke, 1973), *Ziphius cavirostris* (Benirschke and Kumamoto, 1978), *Mesoplodon europaeus* (Árnason *et al.*, 1977) and *M. carlhubbsi* (Árnason *et al.*, 1977). According to Árnason and Benirschke (1973) and Árnason (1974), the 2n = 42 karyotypes in *P. macrorhynchus* and *K. breviceps* do not exhibit clear homologies with the general cetacean karyotype 2n = 44. On the other hand, the 2n = 42 karyotypes of *M. europaeus* and *M. carlhubbsi* are largely in agreement with the general cetacean karyotype (Árnason *et al.*, 1977). Therefore, the chromosomes of *Mesoplodon* species are of great interest when considering karyological evolution in the order Cetacea. However, the chromosomes of only two out of 15 *Mesoplodon* species are known. The Y chromosomes of this genus are also still unknown. The lack of knowledge on the chromosomes of the *Mesoplodon* species is due to the difficulty in collecting living cells from these animals because of their deep sea habitat and in identifying species due to their similar external morphology (Jefferson *et al.*, 2008).

We obtained living cells from males of the Stejneger's beaked whale *M. stejnegeri* and the Hubbs’ beaked whale *M. carlhubbsi* stranded in Japan. The present study provides the first description of the male karyotypes of the *M. stejnegeri* and *M. carlhubbsi*.

A male *Mesoplodon stejnegeri* (NSMT-M 42578), which stranded in Niiya-cho, Sakaiminato-shi, Tottori prefecture, Japan, on March 25, 2014, and a male *M. carlhubbsi* (SNH15011), which stranded in Samani-cho, Hokkaido, Japan, on April 14, 2015, were examined. Both species were identified based on external morphology and tooth shape (Figure 1). The adult male *M. stejnegeri* is characterized by a dark gray body, a head sloping gently down to the beak, and a tusk of which the leading edge is nearly straight and the pointed tip situates almost inline on the superior extension of this leading edge. The adult male *M. carlhubbsi* has a tusk of which the leading edge continues to a shoulder-like curve and the tip is found well behind the leading edge. The whole body is almost dark gray with white portions on the tip of the beak and on a bulged frontal region of the head.

**Figure 1:**
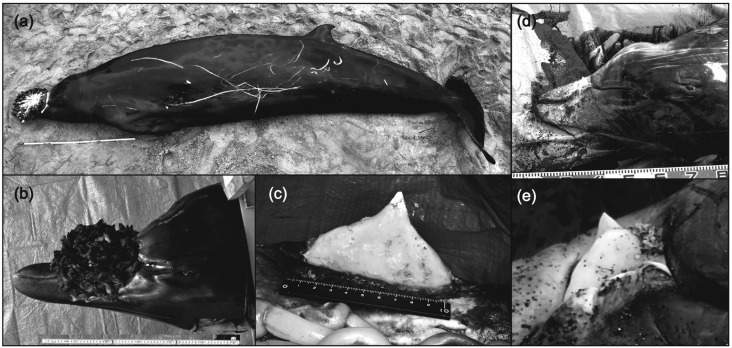
External morphology and tusks of *Mesoplodon stejnegeri* (a, b, and c) and *M. carlhubbsi* (d and e). The tusks of *M. stejnegeri* were observed after removing Conchoderma sp. from them (c).

Small pieces of the intercostal muscle from *M. stejnegeri* and cartilage pieces from the pectoral fin tip of the *M. carlhubbsi* were sampled within 24 hours of their respective deaths and preserved at 4 °C until use. The pieces were cultivated in a culture medium (AmnioMAX^TM^-II Complete medium, Gibco^®^, Life Technology Inc., New York) at 37 °C, 5% CO_2_. The early-passage cells were incubated in hypotonic solution (0.075M KCl) at 37 °C for 18 min after the addition of Colcemid (KaryoMAX^®^ COLCEMID^®^ Solution, Gibco^®^, Life Technology Inc., NY) and incubation at 37 °C for 1–2 h. The cells treated with hypotonic solution were fixed with modified Carnoy's solution (1:3 acetic acid methanol).

C-banding was performed using the barium hydroxide-saline-Giemsa (BSG) method of Sumner (1972). G-banding was also conducted according to the technique of Burgos *et al.* (1986) with some modifications in times. The slide was dried at 95 °C for 23 min. The dried slides were immersed in 0.0125% trypsin (2.5% Trypsin (10X), Gibco^®^, Life Technology) for 7 s, then in 70% ethanol. The slides were treated with 2SSC at 60 °C for 10 min and stained with 4% Giemsa (KaryoMAX^®^ Giemsa Stain Improved R66 Solution “Gurr”,Gibco^®^, Life Technology) for 8 min. Nucleolus organizer regions (NORs) were stained using the one-step method of Howell and Black (1980). We observed a total of 27 cells (conventional karyotype, 18; C-banding, 9) and 17 cells (conventional, 7; C-banding, 4; G-banding, 4; NOR, 2) for *M. stejnegeri* and *M. carlhubbsi*, respectively. The chromosomes were identified as proposed by Levan *et al.* (1964).

The males of *M. stejnegeri* and *M. carlhubbsi* had the same diploid number of chromosomes (2n = 42) but differed in chromosomal morphology (Figures 2 and 3). The karyotype of *M. stejnegeri* comprised 12 metacentric, four submetacentric, two subtelocentric, and two acrocentric autosomal pairs and subtelocentric X and metacentric Y chromosomes. The karyotype of *M. carlhubbsi* comprised 12 metacentric, five submetacentric, and three acrocentric autosomal pairs and subtelocentric X and metacentric Y chromosomes. In both karyotypes, the metacentric autosomes were all small and the submetacentric and subtelocentric autosomes were relatively large. These characteristics are also common throughout the general cetacean karyotype 2n = 44 (Árnason, 1974).

**Figure 2:**
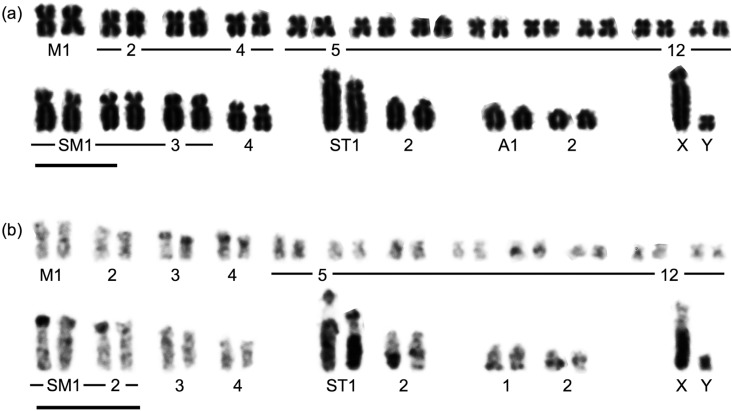
Conventional (a) and C-banding karyotypes (b) of *Mesoplodon stejnegeri*. Bar = 10 μm.

**Figure 3:**
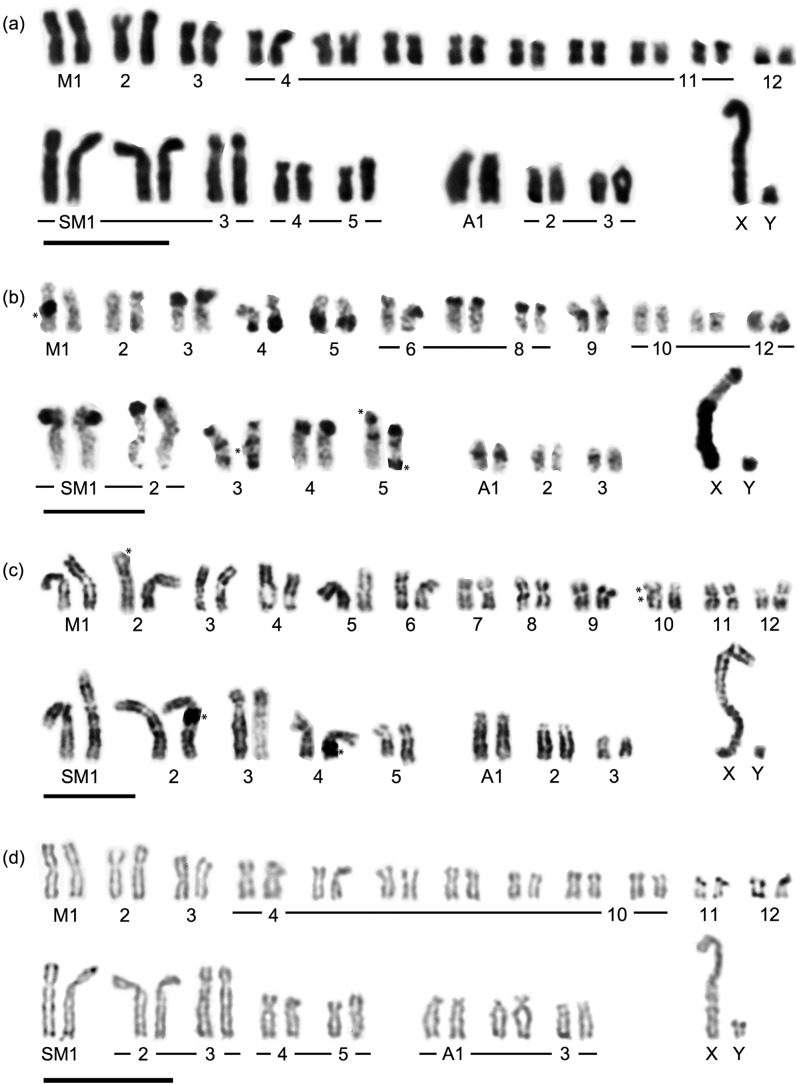
Conventional (a), C-banding (b), G-banding (c), and NOR-banding karyotypes (d) of *M. carlhubbsi*. Bar = 10 μm.

The C-banding karyotypes of both species were characterized by C-heterochromatin accumulation (Figures 2b and 3b). The total lengths of the C-heterochromatic regions of *M stejnegeri* and *M. carlhubbsi* represented 28.4% and 17.8%, respectively, of the total lengths of all chromosomes in the hypothetical female haploid set (autosomes + XX). In another *Mesoplodon* species, *M. europaeus*, the C-banding positive regions occupied 17% of all chromatic regions (Árnason *et al.*, 1977). According to Árnason (1974), in general, the degree of C-heterochromatin accumulation appears to be greater in mysticetes (around 25%) than in odontocetes (12–15%). The degree of C-heterochromatin accumulation in *Mesoplodon* species is similar to that in mysticetes rather than that in odontocetes. Furthermore, notably, *M. stejnegeri* and *M. carlhubbsi* had a large X chromosome with a huge C-block in the long arm. Similar characteristics were also reported in *M. europaeus* and *M. carlhubbsi* by Árnason *et al.* (1977). This characteristic is considered a peculiarity of the *Mesoplodon* species karyotype, because it is not found in other cetaceans, *e.g., Stenella clymene* (Árnason, 1980), *Phocoena phocoena* (Árnason, 1980), *Physeter macrocephalus* (Árnason, 1981a), and *Pontoporia blainvillei* (Heinzelmann *et al.*, 2008). C-banding karyotypes of *M. stejnegeri* and *M. carlhubbsi* also possessed characteristics identical to those of the general cetacean karyotype 2n = 44 described by Árnason (1974): less accumulated C-heterochromatin in the centromeric region and heteromorphism in the C-banding pattern, as shown in ST1 and ST2 of *M. stejnegeri* (Figure 2b) and M4 of *M. carlhubbsi* (Figure 3b). The Y chromosome was small, with its whole body strongly stained in both *M. stejnegeri* and *M. carlhubbsi*. On the other hand, some differences in C-banding pattern were found between *M. stejnegeri* and *M. carlhubbsi*. Whereas *M. stejnegeri* had large C-blocks in ST1 and ST2 (Figure 2b), *M. carlhubbsi* did not (Figure 3b). Interstitial C-bands were found in SM3, SM5, A1, and A3 in *M. carlhubbsi,* but only in A2 in *M. stejnegeri*. Therefore, it is considered that interspecific variation in chromosomal morphology among *Mesoplodon* species appears to be caused by C-heterochromatin accumulation.

The G-banding karyotype of *M. carlhubbsi* exhibited heteromorphisms in SM5 (Figure 3c). The distal G-band positive region of the long arm of SM5 was larger in one of the homologues (Figure 3c). This heteromorphism was in agreement with the C-banding pattern and was found in all cells examined (Figures 3b and c).

The NOR-banding karyotype of *M. carlhubbsi* was obtained on the same slide as that used for the conventional karyotype (Figure 3d). NOR regions were found at the telomeric positions in both the long and short arms of SM1 and at the telomeric positions in the short arms of M11 and M12. Although NORs were not stained for *M. stejnegeri*, a chromosome association was found in one cell, indicating the presence of the NOR regions (Figure 4). A small metacentric autosome and a large subtelocentric autosome (ST1) were attached at the terminal positions of their short arms. It is known that *M. europaeus* has two NOR pairs, one on a large and one on a small autosomal pair (Árnason, 1981b). Therefore, the presence of NORs on a large autosomal pair and on the one or two small autosome pairs would be common throughout *Mesoplodon* species. As mentioned by Árnason (1981b), NORs on the terminal region of the smaller autosomes were also identical to the general cetacean karyotype (2n = 44).

**Figure 4:**
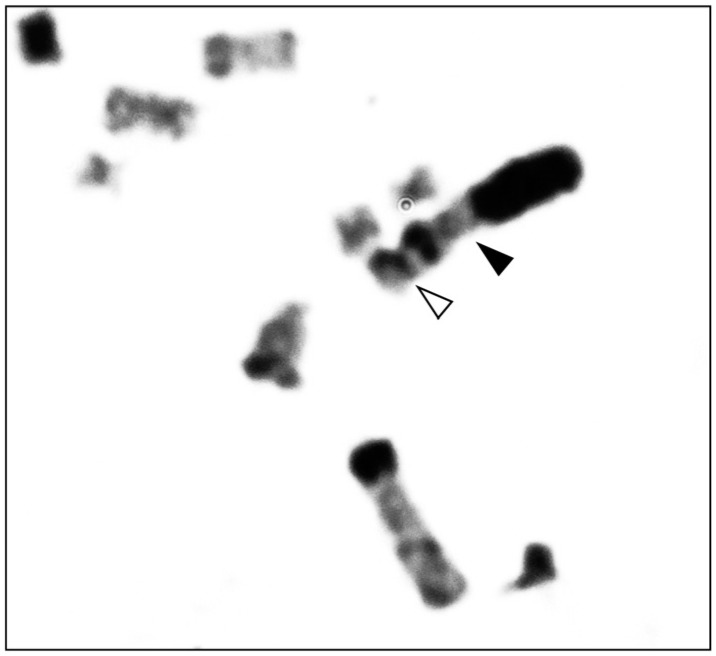
A chromosome association between SM1 (closed arrow) and a small metacentric autosome (open arrow) shown in a metaphase plate of *M. stejnegeri*.

In the present study, the male karyotypes of two whales (*M. stejnegeri* and *M. carlhubbsi*) were clarified. It was confirmed that the karyotypes of *Mesoplodon* species have some peculiarities, and their 2n = 42 karyotype possesses some characteristics identical to those of the general cetacean karyotype 2n = 44. Our findings should help in understanding the cetacean karyological evolution.
